# The relationship between nursing students' attitudes toward artificial intelligence and their creative personality traits

**DOI:** 10.1111/inr.70008

**Published:** 2025-03-12

**Authors:** Kübra Gülırmak Güler, Belgin Şen Atasayar

**Affiliations:** ^1^ Department of Psychiatric Nursing Faculty of Health Sciences Ondokuz Mayıs University Samsun Turkey; ^2^ Department of Surgical Diseases Nursing Faculty of Health Sciences Ondokuz Mayıs University Samsun Turkey

**Keywords:** Artificial intelligence, attitude, creative personality, nursing student

## Abstract

**Aim:**

The relationship between nursing students' attitudes toward artificial intelligence and their creative personality traits was examined in this study.

**Design:**

This study, conducted with 492 nursing students enrolled at a university in Turkey, was designed using a descriptive and relational methodology. The data were gathered through the “Personal Information Form,” the “General Attitude Scale toward Artificial Intelligence,” and the “Creative Personality Traits Scale.”

**Methods:**

The data for the research were gathered from surveys conducted between January 2024 and May 2024.

**Findings:**

The average score for students' attitudes toward artificial intelligence was 74.52 ± 10.29, while the score for creative personality traits was 67.20 ± 10.34. Correlation analysis results indicate a strong relationship between these two factors (*p* < 0.05).

**Conclusions:**

Nursing students' attitudes toward artificial intelligence and creative personality traits are above average.

**Implications for nursing and health policy:**

The development of creativity is crucial for effectively integrating artificial intelligence technologies into nursing practice. Additionally, this research highlights the need for policy development regarding regulations and ethical practices related to using artificial intelligence in healthcare services.

## INTRODUCTION

Artificial intelligence (AI) holds considerable importance in healthcare, especially in areas like early disease detection and personalized treatment approaches, influencing the roles of all healthcare professionals, including nurses. As future healthcare providers, nursing students' perspectives on AI are crucial for embracing these advancements. Therefore, understanding the factors that shape their attitudes toward AI is essential (Abdullah & Fakieh, 2020; Güler & Emirza, 2023). Moreover, creative personality traits can shape how students embrace AI. With its ability to adapt to innovations and develop diverse perspectives, creativity is crucial for nurses in addressing complex health problems (Ahmady & Shahbazi, 2020; Baş et al., 2022; Liu, 2022). Exploring the connection between students' perspectives on AI and creative personality traits may improve their thinking ability and strengthen professional skills. Moreover, it could shed light on the attitudinal and individual differences that may arise while integrating innovative technologies like AI into nursing education, enabling the development of more targeted educational strategies. However, examining the available sources reveals that no specific study has addressed the relationship between these two significant variables, highlighting the necessity and importance of more comprehensive research. This research suggests that individuals with creative personality traits tend to adopt AI technologies more quickly and easily. Furthermore, this relationship may also be relevant to students in other disciplines, such as medicine, design, and education. These findings provide a foundation for exploring the tendencies of students from different professional groups to use AI technologies. Furthermore, they highlight the importance of fostering and leveraging creative personality traits in developing AI technologies among students. The results may provide a profound understanding of integrating AI technologies into health education and practices, potentially contributing to growing national and international health policies, standards, and guidelines for technology's safe and ethical use.

## BACKGROUND

AI advancements in healthcare are transforming nursing practices by enabling faster and more accurate decision‐making in patient care, reducing workloads, and optimizing clinical processes (Labrague et al., 2023; Skiba, 2017a). For instance, AI‐powered systems allow nurses to use their time more efficiently by providing patient monitoring, early warning alerts, and personalized treatment recommendations (Pepito & Locsin, 2019; Ronquillo et al., 2021). However, AI poses disadvantages, such as ethical concerns, privacy breaches, and reduced human touch in patient care (O'Shaughnessy et al., 2023). Therefore, establishing specific standards and ethical policies for using AI in nursing is essential (Akalın & Veranyurt, 2020). Identifying and addressing AI's short‐ and long‐term impacts on nursing students is highly significant for analyzing the factors shaping the profession's future and uncovering its potential contributions to professional development. Analyzing these impacts provides a solid foundation for aligning nursing education programs with modern technologies, enhancing students’ ability to work effectively with AI, and developing new strategies to improve the quality of healthcare services (Booth et al., 2021; İlaslan et al., 2023; O'Connor et al., 2021). Attitudes toward AI can determine how students approach and interact with this new technology (Labrague et al., 2023). Nursing students' attitudes toward AI provide important insights into how they will adopt, use, and integrate this technology with their patients (İlaslan et al., 2023). AI plays a significant role in training competent and effective healthcare professionals for the future while supporting nursing education by providing realistic simulations to enhance clinical skills and improve decision‐making in diagnosing and treating complex diseases (Chang et al., 2022; İlaslan et al., 2023). Additionally, AI‐based tools can help students improve their big data analysis skills and access the latest research more effectively (Skiba, 2017b). This way, nursing students can prepare to lead the future of healthcare by being aware of and effectively utilizing technological innovations (Güler et al., 2024). Creative thinking skills in nursing provide a significant advantage in coping with unforeseen situations and developing effective solutions to complex clinical scenarios (Lafçı & Gülşen, 2022; Liu, 2022). Creative personality traits equip nursing students with the ability to see from different perspectives, effective communication, and collaboration skills, making them more open to understanding and using technology (Baş et al., 2022; Beaird et al., 2018). Creative personality traits facilitate nursing students' quicker adaptation to innovations like AI, playing a crucial role in integrating this technology into educational programs and healthcare services (Ahmady & Shahbazi, 2020; Lafçı & Gülşen, 2022).

National and international studies indicate that students generally possess a high level of awareness regarding AI and display positive attitudes when they recognize its advantages in nursing practice (Ahmed et al., 2022; Chao et al., 2021; Kelly et al., 2022; Labrague et al., 2023; Mousavi Baigi et al., 2023; Yılmaz & Şekeroğlu et al., 2019). However, these attitudes can also be influenced by students' cultural contexts. Cultural differences, healthcare systems, and the educational levels of nurses between countries must be considered when adopting these technologies. For example, while the widespread technological infrastructure in developed countries facilitates faster integration of AI applications, resource constraints and a lack of digital literacy in developing countries may slow down this process. Therefore, nursing, healthcare, and social policies that promote AI use should adopt inclusive approaches to addressing these differences. In Turkey, where this research was conducted, digitalization and technology integration in the healthcare sector are rapidly increasing, providing nursing students with opportunities to interact with AI and other health technologies. Leveraging and adopting these opportunities depends on Turkey's cultural and social dynamics. The general attitude of Turkish society toward technology can influence nursing students' approaches to AI. Therefore, cultural context should not be overlooked when evaluating attitudes toward AI. Current healthcare policies and requirements must be reviewed to implement these technologies effectively. To successfully integrate AI into nursing practices, regulations and standards supporting these technologies should be established in healthcare policies. Furthermore, updating nursing education to align with these technologies is critical for enabling nurses to use AI tools effectively. Expanding the scope and impact of AI applications in nursing practice requires integrating these technologies into nursing education and healthcare policies.

### Study aims

This research aimed to investigate nursing students' perspectives on AI and analyze the association between these views and aspects of creative personality.

### Research questions

- What are nursing students' levels of attitudes toward AI and creative personality traits? What are the mean scores for these two variables?
- Do attitudes toward AI and levels of creative personality traits vary based on the demographic characteristics of nursing students?
- Does a meaningful connection exist between students' attitudes toward AI and their creative personality traits?

## METHOD

### Study design

This study employed a descriptive and correlational design to thoroughly examine participants' attitudes toward AI in alignment with the research goals. The descriptive method allowed for an in‐depth examination of nursing students' current attitudes and perceptions, while the correlational analysis provided insights into how these attitudes relate to various variables.

### Inclusion and exclusion criteria

#### Inclusion criteria

To be eligible for participation in the study, consenting individuals were reuired to be between the ages of 18 and 65 and actively pursuing their education in the nursing department during the 2023–2024 academic year. Additionally, participants were also required to have access to a phone, tablet, or computer to complete the survey, administered via Google Forms.

#### Exclusion criteria

Individuals who lacked the necessary language or technical skills to complete the survey, such as those who required assistance in understanding its language, were excluded from the study. Additionally, those who had recently participated in a similar study were not included. Furthermore, individuals who explicitly declined to participate were also excluded from the research.

### Setting

The research was conducted within a university's nursing department in Turkey's Black Sea Region to ensure efficient data collection and meet the desired sample size. The selection of this department provided a suitable environment for the study and allowed for the efficient and timely execution of the research process. The selected nursing department offers a four‐year undergraduate program, providing students with an intensive learning experience combining theoretical and practical components. The curriculum covers various subjects, from foundational sciences to professional healthcare applications, which fosters students' academic and professional development. Students gain practical experience by integrating theoretical knowledge with clinical applications, making this population particularly suitable for exploring attitudes toward AI and creative personality traits. This context makes the department an ideal setting for investigating the research topics, as it provides insights into how nursing students interact with advanced technologies and how their creativity and attitudes develop during their education.

### Participants

The study encompassed students enrolled in the nursing department of a university located in the Central Black Sea Region of Turkey during the 2023–2024 academic year, covering all academic levels from first to fourth year. Out of 597 students, those who satisfied the inclusion criteria were chosen as the study sample. Data were collected between January 2024 and May 2024 using Google Forms, with participants taking approximately 10 minutes to complete the survey. Google Forms was chosen as the data collection method due to its accessibility and efficiency in managing the process.

A random selection approach was employed to guarantee that the sample accurately represented the population's characteristics. This method provided each student an equal opportunity to be included, enhancing the likelihood that the sample reflected the larger population. A list of enrolled nursing students was obtained to define the study population, and those meeting the inclusion criteria were randomly selected. A pre‐screening process based on established criteria ensured eligibility, leading to the exclusion of two students who still needed to meet the requirements. Of the 510 eligible students invited, 18 declined participation, resulting in a final sample meeting all criteria.

As shown in Table 1, most participants were women, and almost all were single (99.4%). The distribution of students across academic years was as follows: 25.6% in the first year, 26.2% in the second year, 23.6% in the third year, and 24.6% in the final year. Most students (89.2%) reported prior familiarity with AI. Interest in creative activities, often linked to creative personality traits, was expressed by 59.4% of the students. Additionally, 90% indicated using online resources to acquire information about AI, and 77.6% assessed their technological proficiency as moderate.

**TABLE 1:** Sociodemographic characteristics of nursing students.

Features	*n*	%
Gender		
Female	273	55.5
Male	219	44.5
Marital status		
Single	489	99.4
Married	3	0.6
Classroom		
1st claass	126	25.6
2nd class	129	26.2
3rd class	116	23.6
4th class	121	24.6
Previous training or experience in AI		
Yes	439	89.2
No	53	10.8
Creative work (painting, music, writing, etc.)		
Yes	292	59.4
No.	200	44.6
Resources for learning about AI		
Books	27	5.5
Makeover	22	4.5
Internet resources	443	90.0
Other		
Level of technological aptitude		
Low level	57	11.6
Moderate	382	77.6
High level	53	10.8
Total	492	100
	Min–max; *X*‾ ± SD	
Average age	17–37; 20.9 ± 2.3	

Abbreviations: min–max, minimum–maximum; *n*, sample size; Sd, standard deviation; *X*‾, mean.

### Variables of the study

**Dependent variables**: Attitude levels toward AI, creative personality traits

**Independent variables**: Gender, age, marital status, class, experience in AI sources used to obtain information and level of predisposition to technology.

### Data collection tools

#### Personal information form

The researcher developed this survey to determine students' sociodemographic characteristics based on a comprehensive literature review. The form consists of a total of 8 questions. These questions cover gender, marital status, age, prior education or experience related to AI, creative activities (such as painting, music, and writing), sources used to acquire knowledge about AI, and the level of affinity for technology. The content of the form was shaped using data and recommendations derived from previous studies (Akalın & Veranyurt, 2020; Aslan & Subaşı, 2022; İlaslan et al., 2023).

#### General attitudes toward artificial intelligence scale (GAAIS)

The GAAIS is a 20‐item scale designed to evaluate attitudes toward AI, with its validity and reliability confirmed by a study conducted in 2022 (Kaya et al., 2022). The scale includes two subcategories: “Positive Attitude” (12–60 points) and “Negative Attitude” (8–40 points), with a total score standardized between 0 and 100, where greater scores reflect more favorable attitudes. The Cronbach's alpha values of the scale range from 0.82 to 0.88, with reliability levels of 0.77 for positive attitudes and 0.83 for negative attitudes. In this study, Cronbach's alpha values for the total score, positive attitude, and negative attitude were calculated as 0.865, 0.890, and 0.875, respectively.

#### Creative personality traits scale (CPTS)

The CPTS, developed by Şahin and Danışman (2017), measures individuals' creative personality traits influenced by cultural factors. The scale consists of 17 items divided into four dimensions: goal orientation, intrinsic motivation, self‐confidence, and risk‐taking. Participants rate their responses using a 5‐point Likert scale from “strongly disagree” to “strongly agree.” The total score ranges from 17 to 85, with subscale scores detailed as follows: goal orientation and intrinsic motivation (5–25), risk‐taking (4–20), and curiosity (3–15). The scale was validated through confirmatory factor analysis, with fit indices of χ^2^/df = 2.332, RMSEA = 0.04, SRMR = 0.04, GFI = 0.97, and CFI = 0.95. Şahin and Danışman (2017) reported Cronbach's alpha values of 0.60–0.65 for subscales and 0.67 for the total scale. In this study, the total Cronbach's alpha was calculated as 0.875, with subscale values ranging from 0.752 to 0.894, confirming the reliability and applicability of the CPTS in this context.

### Data collection

**FIGURE 1:**
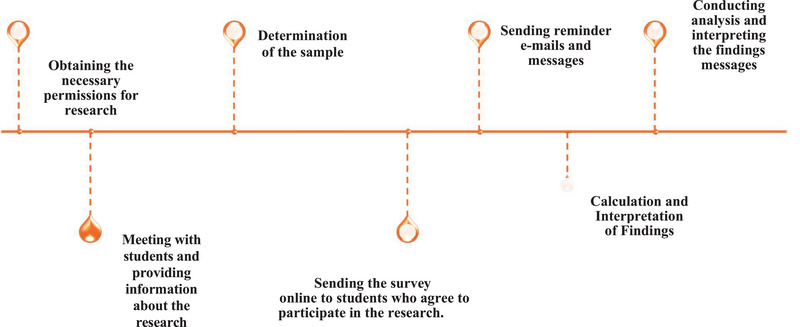
Research process timeline.

The research data were collected over five months, from January 2024 to May 2024. During this time, participants were asked to complete the Personal Information Form, GAAIS, and CPTS questionnaires. Before the data collection process began, researchers visited classrooms to provide students with detailed information about the study. These informational sessions explained the research's purpose, content, methods, and confidentiality principles. Students interested in participating were invited to one‐on‐one meetings, during which their questions were answered, and the research process was explained in detail. Verbal consent was obtained from participants, emphasizing the voluntary nature of their participation. To facilitate access, as all students had institutional Gmail accounts, surveys were prepared using Google Forms. Researchers accessed the students' email addresses through a list provided by the department secretariat. Survey links were sent to students' institutional email addresses and, for those who did not use email, via class WhatsApp groups. A week later, reminders and WhatsApp messages were sent to non‐responders to increase participation rates. Measures were implemented to ensure that each participant could respond only once (Figure 1).

At the start of the surveys, participants were asked to provide electronic consent to reaffirm their voluntary participation. Ensuring participant confidentiality and data security remained a top priority throughout the research process. Additionally, the responsible researcher's contact information was shared so participants could address any questions or concerns about the study. These measures ensured that participants felt secure and comfortable engaging in the process and could easily reach the researcher.

The research data were collected through online surveys. This method provided participants with time and location flexibility, allowing them to respond conveniently. Additionally, responses were recorded instantly, enabling researchers to receive quick feedback and perform real‐time data analysis. Furthermore, online surveys reduced paper usage, contributing to environmental sustainability. In conclusion, online surveys supported a more efficient research process, reduced environmental impact, and facilitated participant engagement.

### Research ethics

Great care was taken regarding ethical principles throughout the study. Participants were informed that all responses would be analyzed anonymously and that individual identities could not be determined under any circumstances. This assurance was a fundamental principle to encourage voluntary participation and alleviate participants' concerns. Since the researchers were also faculty members at the same institution, specific measures were taken to ensure participants felt free of pressure or obligated to participate. Both in the written consent process and through individual communication, participants were told that their participation would not affect their academic performance, exam results, course grades, or any other evaluations. This was repeatedly emphasized to address any potential concerns. The survey forms and other data collection tools did not request identifying information. Strict measures were implemented during data storage and processing to ensure confidentiality, with access restricted to authorized personnel and security protocols applied. These steps were carefully planned and rigorously enforced to ensure participants could engage in the study voluntarily, without any concerns or hesitation, while maintaining the highest ethical standards throughout the research process.

## RESULT

### Total and subscale mean scores of GAAIS and CPTS

According to Table 2, the total score of GAAIS was 74.52 ± 10.29. The positive attitude toward AI subdimension score, which is one of the subdimensions of this scale, was 46.81 ± 7.20, and the negative attitude subdimension score was 27.71 ± 6.30. When the CPTS was analyzed, it was found that the scale's total score was 67.20 ± 10.34. When the subdimension were analyzed, the mean score for the goal orientation subdimension was determined to be 18.02 ± 5.61, the average score for the intrinsic motivation subdimension was 21.27 ± 6.30, the average score for the self‐confidence subdimension was 11.12 ± 3.05, and the average score for the risk‐taking subdimension was 16.78 ± 3.05.

**TABLE 2:** Mean scores of the nursing students in the general attitude toward artificial intelligence and creatice personality traits scale.

Scales	Scale total score and subdimensions	Min–max	X‾ ± SD
General attitude toward artificial intelligence scale (GAAIS)	General attitude toward artificial intelligence scale Total score	37–97	74.52 ± 10.29
	Positive attitude toward artificial intelligence	18–60	46.81 ± 7.20
	Negative attitude toward artificial intelligence	8–40	27.71 ± 6.30
Creative Personality Traits Scale (CPTS)	Creative Personality Traits Scale Total Score	41–85	67.20 ± 10.34
	Goal orientation	5–25	18.02 ± 5.61
	Intrinsic motivation	13–25	21.27 ± 6.30
	Self‐confidence	3–15	11.12 ± 3.05
	Risk‐taking	8–20	16.78 ± 3.05

Abbreviations: Min–max, minimum–maximum; SD, standard deviation; *X*‾, mean.

### Comparison of GAAIS and CPTS scores according to sociodemographic characteristics of nursing student

Table 3 shows significant differences in the GAAIS total score, positive attitude subscale, and CPTS intrinsic orientation subscale scores based on gender (*p* < 0.05). Significant differences were also identified between grade levels and the negative attitude subscale, total creative personality traits, intrinsic motivation, and risk perception subscale scores (*p* < 0.05). Participation in creative activities significantly impacts GAAIS total and subscale scores and the subdimensions of creative personality traits such as goal orientation, intrinsic motivation, and risk perception (*p* < 0.05). While a significant relationship was found between technological predisposition and GAAIS total and subscale scores (*p* < 0.05), no significant relationship was observed between methods of obtaining AI‐related information and scale scores (*p* > 0.05).

**TABLE 3:** Distribution of the GAAIS and CPTS of the nursing students based on their sociodemographic characteristics.

Sociodemographic	General Attitude Scale Toward Artificial Intelligence						Creative Personality Traits Scale									
Demographic characteristics	Total score of the General Attitude Scale Toward Artificial Intelligence		Positive attitude towards artificial intelligence		Negative attitude toward artificial intelligence		Creative Personality Traits Scale total score		Goal orientation		Intrinsic motivation		Self‐confidence		Risk‐taking	
	X‾ ± SD	*p*	X‾ ± SD	*p*	X‾ ± SD	*p*	X‾ ± SD	*p*	X‾ ± SD	*p*	X‾ ± SD	*p*	X‾ ± SD	*p*	X‾ ± SD	*p*
Gender																
Female	67.97 ± 10.30	**0.003** *	46.07 ± 6.62	**0.004** *	27.26 ± 6.01	0.103	97.97 ± 10.30	0.059	18.19 ± 5.77	0.317	21.64 ± 2.28	**0.000** *	11.13 ± 2.98	0.094	17.00 ± 2.96	0.072
Male	76.00 ± 10.79		47.73 ± 7.78		28.26 ± 6.60		66.24 ± 10.32		17.81 ± 5.40		20.81 ± 2.78		11.10 ± 3.15		16.51 ± 3.16	
Classroom																
1st grade	74.95 ± 8.75	0.286	46.59 ± 7.02	0.872	28.35 ± 5.70	**0.039** *	70.01 ± 9.63	0.001**	19.48 ± 5.52	**0.000** **	21.88 ± 2.27	**0.022** **	11.49 ± 2.64	0.055	17.15 ± 2.93	**0.014** **
2nd grade	75.67 ± 10.60		47.30 ± 7.29		28.37 ± 6.81		65.76 ± 9.09		18.14 ± 5.12		20.88 ± 2.56		10.53 ± 3.13		16.20 ± 2.95	
3rd grade	74.57 ± 8.74		46.93 ± 5.92		27.63 ± .585		67.68 ± 11.11		18.00 ± 5.72		21.00 ± 2.70		11.38 ± 3.22		17.28 ± 3.10	
4th grade	72.80 ± 12.47		46.39 ± 8.37		26.40 ± 6.82		65.34 ± 10.95		16.39 ± 5.72		21.31 ± 2.56		11.10 ± 3.15		16.52 ± 3.14	
Presence of experience in artificial intelligence																
Yes	74.73 ± 10.39	0.216	46.81 ± 7.29	0.883	27.92 ± 6.24	**0.018** *	67.19 ± 10.39	0.998	17.89 ± 5.66	0.186	21.20 ± 2.59	0.100	11.23 ± 3.05	**0.011** *	16.84 ± 3.08	0.115
No.	72.81 ± 9.30		46.83 ± 6.50		25.98 ± 6.54		67.32 ± 10.01		19.07 ± 5.12		21.83 ± 2.10		10.16 ± 2.96		16.24 ± 2.79	
Status of doing creative work																
Yes	69.78 ± 8.41	**0.000** *	44.70 ± 5.99	**0.000** *	25.08 ± 5.07	**0.000** *	67.86 ± 9.56	0.402	18.70 ± 5.66	**0.049** *	20.57 ± 1.90	**0.000** *	11.06 ± 2.82	0.480	17.51 ± 2.96	**0.000** *
No.	76.80 ± 10.34		47.82 ± 7.51		28.97 ± 6.44		66.86 ± 10.69		17.69 ± 5.56		21.61 ± 2.75		11.14 ± 3.16		16.43 ± 3.04	
Sources used to obtain information																
Books	73.85 ± 10.56	0.611	44.29 ± 8.57	0.198	29.55 ± 5.80	0.262	69.59 ± 10.71	0.265	18.7 ± 6.64	0.331	22.07 ± 1.99	0.142	11.37 ± 3.09	0.858	17.4 ± 2.81	0.265
Articles	76.50 ± 10.56		48.40 ± 5.17		28.09 ± 7.42		69.68 ± 9.78		19.63 ± 4.52		21.59 ± 2.08		11.00 ± 2.96		17.45 ± 2.32	
Internet resources	74.46 ± 10.27		46.88 ± 7.17		27.58 ± 6.26		6.93 ± 10.33		17.90 ± 5.59		21.20 ± 2.59		11.11 ± 3.06		16.71 ± 3.10	
Level of technological aptitude																
Low level	76.07 ± 9.85	**0.027** **	47.61 ± 7.07	**0.038** **	28.46 ± 5.85	**0.000** **	67.31 ± 9.89	0.158	18.72 ± 5.24	0.217	21.17 ± 2.48	0.103	11.05 ± 2.98	0.071	16.36 ± 3.11	0.104
Moderate	4.06 ± 10.38		46.57 ± 7.23		27.48 ± 6.41		67.17 ± 10.48		17.81 ± 5.71		21.30 ± 2.57		11.14 ± 3.08		16.90 ± 3.03	
High level	75.67 ± 10.90		47.43 ± 6.92		28.24 ± 7.66		65.77 ± 11.26		17.32 ± 6.38		21.07 ± 2.50		10.28 ± 3.49		17.09 ± 2.67	

	***r* values**		***r* values**		***r* values**		***r* values**		***r* values**		***r* values**		***r* values**		***r* values**	
Average age	−0.041	0.369	0.034	0.449	−0.083	0.065	−0.142	**0.002** *	−0.157	**0.000** *	−0.128	0.004	−0.050	0.265	0.009	0.846

Abbreviations: SD, Standard deviation; X‾, mean.

* *p* < 0.05; Mann−Whitney *U* test (Z‐table value) was used for the comparison of two independent groups.

** *p* < 0.05; Kruskal−Wallis *H* test (χ^2^‐table value) was used to compare three or more independent groups that did not have a normal distribution.

### Relationship between the GAAIS scale and CPTS

Table 4 reveals a significant and strong positive relationship between GAAIS and CPTS (*r* = 0.930, *p* < 0.05). This indicates that students' creative personality traits increase and their attitudes toward AI become more positive. Additionally, a weak negative relationship was found between creative personality traits and the negative attitude subscale of GAAIS (*r* = −0.167, *p* > 0.05), suggesting that negative attitudes toward AI tend to decrease as creative personality traits grow. These findings emphasize the importance of encouraging creativity to enhance students' ability to adapt to AI and develop positive attitudes toward this technology.

**TABLE 4:** General Attitude Toward Artificial Intelligence Scale and Creative Personality Traits Scale total mean score correlation.

Scales and subscales	GAAIS total score (*r*)	Positive attitude (*r*)	Negative attitude (*r*)	Creative personality traits scale total score (*r*)	Goal orientation (*r*)	Intrinsic motivation (*r*)	Self‐confidence (*r*)	Risk‐Taking (*r*)
GAAIS total score	1							
Positive attitude	**0.774** **	1						
Negative attitude	**−0.730** **	**0.167** **	1					
Creative personality traits scale total score	**0.930** *	0.019	**−0.115** *	1				
Goal orientation	**0.133** **	0.039	**0.170** **	**0.813** **	1			
Intrinsic motivation	0.081	**0.101** *	0.014	0.667	**0.545** **	1		
Self‐confidence	0.038	0.039	0.079	**0.719** **	**0.300** **	**0.265** **	1	
Risk‐taking	**0.980** *	**0.128** **	−0.022	**0.584** **	**0.156** *	**0.107** *	**0.678** **	1

*p* < 0.05; Spearman's correlation coefficient was used to examine the correlation between two quantitative variables that did not have a normal distribution. *r* = Correlation coefficient, Spearman correlation test.

* *p* < 0.05.

** *p* < 0.001.

## DISCUSSION

This study identified a significant relationship between nursing students' GAAIS and CPTS, highlighting that creative personality traits are crucial in shaping their attitudes toward AI (Table 4). Creativity provides nursing students with numerous advantages in both personal and professional development. Research indicates that creativity enhances problem‐solving abilities, supports clinical decision‐making, and fosters innovative solutions to complex healthcare challenges (Ahmady & Shahbazi, 2020; Baş et al., 2022; Liu et al., 2020). Furthermore, creative thinking skills contribute to emotional resilience and empathy by strengthening coping mechanisms (Fatma et al., 2020; Liu et al., 2020). Encouraging creativity enables students to adapt more efficiently and seamlessly to both individual and team‐based work settings. Technological advancements, particularly in AI, are transforming the workflows and dynamics of nursing practice. Understanding the interplay between creative personality traits and attitudes toward AI is, therefore, essential for designing educational programs that address the evolving needs of the nursing profession. The findings of this research align with international studies on nursing students' perceptions of AI. Research conducted in countries such as Japan and the United States shows that students generally have positive attitudes toward AI (Ho et al., 2023; Labrague et al., 2023; Lukić et al., 2023). These findings highlight the global importance of integrating AI into nursing education. While this study reflects the influence of Turkish culture, international research that considers cultural diversity is essential to understanding the broader impact of AI perception and adoption. Comparative studies in different cultural contexts will evaluate the applicability of these findings and provide a more comprehensive roadmap for integrating AI technologies into nursing education.

It is widely recognized that culture plays a crucial role in successfully adopting technological innovations in nursing education. However, a review of the literature indicates that while there are studies addressing nursing students' attitudes toward AI, the number of such studies remains relatively limited (Ahmed et al., 2022; Chao et al., 2021; Kelly et al., 2022; Labrague et al., 2023; Mousavi Baigi et al., 2023; Yılmaz & Şekeroğlu, 2019). The research explores the connection between nursing students' views on AI and traits linked to creative personality. The findings indicate that various factors, including individual personality characteristics, can shape attitudes toward AI. The link between creative personality traits and AI perceptions offers valuable insight into how this technology could be leveraged more effectively in healthcare settings. This research contributes significantly to understanding how future healthcare professionals might integrate such technologies by examining nursing students' attitudes toward AI and their creative thinking capabilities. Supporting creative traits among students may enhance their engagement with AI, fostering innovative problem‐solving and effective use of technology. Creativity is vital in developing new ideas, addressing challenges, and utilizing technology efficiently. Encouraging creative personality traits in nursing students could improve their attitudes toward AI, enhancing patient care and higher‐quality healthcare services. Consequently, the findings of this study hold substantial importance in shaping educational strategies and healthcare practices.

The study's findings reveal that nursing students hold a notably positive attitude toward AI, averaging 74.52 ± 10.29 (Table 2). This result is consistent with prior research exploring nursing students' perspectives on AI. For example, a study by Labrague et al. (2023) found that nursing students demonstrated high awareness of the use of AI. Similarly, Lukićet al. (2023) reported that nursing students had above‐average attitudes toward AI, with a mean score of 64.5 ± 11.7. Additionally, Yılmaz and Şekeroğlu (2019) identified that 47.4% of students were familiar with AI, aligning with the present study's results. Other research in the literature also indicates that nursing students tend to adopt more positive attitudes toward AI when they view it as a practical and advantageous tool in their field (Ahmed et al., 2022; Chao et al., 2021; Gado et al., 2022; Kelly et al., 2022; Mousavi Baigi et al., 2023; Sit et al., 2020; Yılmaz & Şekereoğlu, 2019). However, contrasting perspectives have also been noted. For instance, Abdullah and Fakieh (2020) found that healthcare professionals in Saudi Arabia expressed apprehensions about integrating AI into healthcare settings, fearing job displacement. Similarly, Taryudi et al. (2022) highlighted concerns among Indonesian nurses that robots might eventually replace their roles, potentially undermining their professional skills.

The study findings determined that students' attitudes toward AI were influenced by gender, previous creative activities, and their predisposition to technology (*p* < 0.05, Table 3). Like these findings, some studies reveal that attitudes toward AI may differ between genders (Bisdas et al., 2021; Chen et al., 2022; European Commission, 2017; Lukić et al., 2023; O'Shaughnessy et al., 2023; Pinto Dos Santos et al., 2019; Zhang & Dafoe, 2019). This may be due to the interaction of various factors between these variables. For example, gender may be influenced by social norms, cultural factors, and individual experiences, which may shape attitudes toward AI. Previous creative work may contribute to individuals' development of creativity and problem‐solving skills, which in turn may influence attitudes toward AI. The level of technological aptitude may be associated with individuals' skills and habits of using technology, which may also affect attitudes toward AI. Therefore, the reason why students' attitudes toward AI are influenced by gender, the status of doing creative work, and the level of technological aptitude are that these factors come together to shape individuals' perceptions and attitudes.

The connection between creativity and attitudes toward AI highlights how individuals' levels of creativity interact with their perceptions of AI. Creativity involves thinking innovatively and developing unique solutions, while AI refers to machines demonstrating human‐like intelligence and learning capabilities. This study's findings indicate that nursing students exhibit above‐average creative personality traits, with a mean score of 67.20 ± 10.34, suggesting a strong inclination toward creativity (Table 2). These results align with similar studies in the literature. One study found that nursing students exhibited high levels of creative personality traits. Similarly, another study reported that nurses generally demonstrated moderate levels of creativity. However, a survey conducted with senior nursing students in Taiwan concluded that students had low levels of creativity (Baş et al., 2022; Lafçı & Gülşen, 2022; Liu et al., 2020). These results imply that fostering creative personality traits can positively influence students' attitudes toward AI. Creativity can serve as a valuable tool in addressing complex challenges and generating innovative ideas, potentially enhancing the merger of AI into nursing education and practice. The study revealed significant differences in creative personality traits across class levels (*p* < 0.05, Table 3). It was noted that students' grade levels and age impacted their creative personalities. As students progress in age and grade, their experiences, educational exposure, and social contexts evolve. For instance, younger students may still develop their creative personality traits, while the varying learning content at different grade levels can influence their creativity. Likewise, in more advanced grades, students' creativity may be enhanced because they have gained more experience and have encountered more complex subjects. Therefore, many factors, such as life experiences, learning processes, and social interactions, may impact students' creative personality levels, influencing their grades and age. These findings demonstrate that various factors significantly influence nursing students' attitudes toward AI.

## RECOMMENDATIONS FOR NURSING EDUCATORS AND POLICYMAKERS

It is recommended to establish common international standards and guidelines for integrating AI into nursing education, fostering international collaboration, and sharing best practices to ensure consistency and effectiveness. Additionally, the development of ethical and behavioural policies is essential to guide the responsible use of AI technologies in nursing education. Ongoing research and evaluation should be supported to monitor AI's impact on education, ensuring that adequate resources and funding are available for its sustainable implementation. Furthermore, students should be given opportunities to engage with AI and digital technologies through scenario‐based learning, projects, and research initiatives that promote creativity and innovation.

## LIMITATIONS AND STRENGTHS OF THE STUDY

### Strengths

The literature review indicates the need for descriptive studies on AI in nursing, both internationally and nationally. This research fills this gap by examining nursing students' views on AI and identifying the factors that shape their attitudes. It also explores the relationship between creative personality traits and AI attitudes, highlighting the role of creativity in fostering AI development. The study provides a solid cross‐sectional, descriptive, and correlational dataset. The measurement tools used have been validated for reliability and accuracy, and the findings offer recommendations to increase AI awareness and promote creativity in nursing education. This study contributes to understanding the relationship between AI and creativity among nursing students in Turkey and emphasizes the impact of Turkey's cultural context on these attitudes. However, recognizing the importance of an international perspective, the need for studies considering cultural diversity is also evident. In this context, research in different cultural settings is crucial to understanding the broader impacts of AI perception and adoption, and the results may contribute to the development of effective educational strategies and policy frameworks on a global scale. Additionally, the findings of this study may serve as a guide for students from other professional backgrounds.

### Limitations

This study has several noteworthy limitations. First, the cultural and social environments in which the research was conducted may need to be revised to allow the applicability of the findings. Different social and professional settings could shape the connection between nursing students' creative personality traits and their attitudes toward AI. Examining these varied contexts further could enhance the generalizability and global relevance of the results. Moreover, students' participation levels and response rates might have influenced the outcomes. To achieve more comprehensive and applicable insights for incorporating AI into nursing education, future research should investigate how diverse cultural and social factors affect these relationships.

## IMPLICATIONS FOR NURSING AND HEALTH POLICY

This research highlights the significant connection between nursing students' creative personality traits and their attitudes toward AI, emphasizing that fostering creativity is crucial for facilitating the integration of AI into clinical practice in the context of nursing education and policy development. However, establishing clear policies and guidelines is essential to ensure AI's effective and ethical use. The study advocates adopting educational policies that encourage creative thinking among nursing students and the development of protocols regulating the responsible use of AI. This research contributes to global nursing knowledge and practices by incorporating international comparisons and contextual analyses. Its comprehensive approach underscores the study's importance, aiming to enhance the benefits of AI in nursing education and provide guidance for educators and policymakers.

## CONCLUSION

This research reveals a notable relationship between nursing students' perspectives on AI and aspects of their creative personality. These results emphasize the importance of fostering creative personality traits to enhance students' ability to utilize AI technologies effectively. Furthermore, this relationship offers valuable guidance for shaping international nursing education policies. Integrating AI technologies into nursing education can assist students in enhancing their innovative thinking and problem‐solving abilities, creating a positive global impact on healthcare services. The study suggests developing creative strategies to support AI's broader and more effective use in nursing education. These strategies aim to design educational content that enables students to gain familiarity with AI technologies and incorporate them into their professional practices. AI‐supported tools such as simulation‐based training, big data analysis, and patient‐centered scenarios can enhance students' clinical competencies and facilitate the adaptation of future healthcare professionals to these technologies. Moreover, educational models should be flexible and aligned with international standards to address the diverse needs of nurses in different countries. Such an approach could facilitate the widespread adoption of AI applications, enhance service quality, and contribute to achieving equity in healthcare systems on a global scale. Students' positive attitudes toward AI and their creative thinking skills can drive the widespread adoption of innovative practices and enhance efficiency within healthcare systems. In conclusion, this study provides recommendations that can significantly contribute to healthcare education policies and global healthcare services by integrating AI into nursing education.

## AUTHOR CONTRIBUTIONS

*Study design*: Kübra Gülirmak Güler *Data collection*: Kübra Gülirmak Güler and Belgin Sen Atasayar. *Data analysis*: Kübra Gülirmak Güler. *Study supervision*: Kübra Gülirmak Güler and Belgin Sen Atasayar. *Manuscript writing*: Kübra Gülirmak Güler. *Critical revisions for important intellectual content*: Kübra Gülirmak Güler and Belgin Sen Atasayar.

## CONFLICT OF INTEREST STATEMENT

The authors declare no conflicts of interest.

## ETHICAL APPROVAL

Ethical approval was obtained from the Ondokuz Mayis University Scientific Research Ethics Committee approval (Date: 29/12/2023, Number: 2023/1133).

## Data Availability

The data supporting this study's findings are available on request from the corresponding author. The data are not publicly available due to privacy and ethical restrictions.
